# Family Structure, Family Transitions, and Child Overweight and Obesity: Comparing Australia, the United Kingdom, and the United States

**DOI:** 10.3390/children11060693

**Published:** 2024-06-06

**Authors:** Sadie A. Slighting, Kirsten Rasmussen, Mikaela J. Dufur, Jonathan A. Jarvis, Shana L. Pribesh, Alyssa J. Alexander, Carolina Otero

**Affiliations:** 1 Department of Sociology, Brigham Young University, 2008 JFSB, Provo, UT 84602, USA; sadie.slighting@gmail.com (S.A.S.); kirsten.rasmussen2153@gmail.com (K.R.); jonathan_jarvis@byu.edu (J.A.J.); 2 Department of STEM Education & Professional Studies, Old Dominion University, 2300A Education Building, Norfolk, VA 23529, USA; SPribesh@osu.edu; 3 Department of Sociology, University of British Columbia, AnSo-2220, Vancouver, BC V6T 1Z2, Canada; alyssa.alexander@ubc.ca; 4 United Way of Salt Lake City, 257 E 200 S, Salt Lake City, UT 84111, USA; carolina.otero@uw.org

**Keywords:** childhood overweight, childhood obesity, family structure, family trajectories, single parents, cohabitation, divorce, remarriage, stepparents

## Abstract

Growing rates of childhood obesity globally create concern for individuals’ health outcomes and demands on health systems. While many policy approaches focus on macro-level interventions, we examine how the type of stability of a family structure might provide opportunities for policy interventions at the micro level. We examine the association between family structure trajectories and childhood overweight and obesity across three Anglophone countries using an expanded set of eight family structure categories that capture biological relationships and instability, along with potential explanatory variables that might vary across family trajectories and provide opportunities for intervention, including access to resources, family stressors, family structure selectivity factors, and obesogenic correlates. We use three datasets that are representative of children born around the year 2000 and aged 11 years old in Australia (*n* = 3329), the United Kingdom (*n* = 11,542), and the United States (*n* = 8837) and nested multivariate multinomial logistic regression models. Our analyses find stronger relationships between child overweight and obesity and family structure trajectories than between child obesity and obesogenic factors. Children in all three countries are sensitive to living with cohabiting parents, although in Australia, this is limited to children whose parents have been cohabiting since before their birth. In the UK and US, parents starting their cohabitation after the child’s birth are more likely to have children who experience obesity. Despite a few differences across cross-cultural contexts, most of the relationship between family structures and child overweight or obesity is connected to differences in families’ access to resources and by the types of parents who enter into these family structures. These findings suggest policy interventions at the family level that focus on potential parents’ education and career prospects and on income support rather than interventions like marriage incentives.

## 1. Introduction

Rising standards of living across the globe are generally a cause for celebration, but with these improvements have come a public health challenge—rising obesity rates among children [1]. This pattern is of concern because children who are overweight or obese risk of a number of negative outcomes, potentially including physical health issues, such as diabetes [2,3], cardiometabolic disorders [4], or kidney disease [5]; becoming obese adults and experiencing health problems in adulthood [6,7,8]; or experiencing mental health or social challenges such as stigmatization and discrimination [9,10,11].

Many public policy approaches to these issues focus on macro-societal interventions, such as taxing high-calorie food or drinks or bans on fast food advertising directed at children [12]. We propose a different approach here. Given the primacy of family relationships in young children’s lives, the concurrent pattern of rising numbers of children being raised outside of a “traditional” family structure with two married, biological parents [13] and previous evidence of a higher prevalence of obesity among children living in different family structures [14,15,16], we examine whether children exposed to different family structures and trajectories have patterns of overweight and obesity that might be addressed by policies that address micro-level family resources and behaviors. Our approach includes examining these patterns in three Anglophone countries (Australia, the United Kingdom [UK], and the United States [US]) that have widely varying existing social safety nets for families, which may provide evidence for the idea that a focus on family resources and choices may be a useful avenue for preventing childhood overweight and obesity.

Extant research demonstrates relationships between family structure and a variety of child outcomes, such as behavior problems, educational attainment, and both physical and mental health [17,18]. A large body of research demonstrates associations between living in single-parent, stepparent, and cohabiting families and poorer average outcomes for children [14,15,19,20,21]. However, recent research suggests that these associations may be spurious, connected instead to circumstances and actors that vary across family structures. For instance, resources vary by family structure; single-mother households experience greater economic instability than households with two married parents [22].

A similar debate persists as to how family processes and family structure might influence child obesity risk. Some research has shown an association between family structure and childhood obesity [23], with children living with two parents being less at-risk for obesity than children living with single parents. Such findings are perhaps unsurprising given the robust literature that finds strong associations between living in two-parent families and a variety of health- and achievement-related outcomes [24,25].

Competing perspectives, however, suggest that resources, such as income and education, and parenting behaviors, such as control or neglect, are more important in understanding child obesity and that family structure serves merely as a proxy for these factors [26,27,28]. For example, resources, or lack thereof, play a role in child obesity, in that children who have less access to resources may be at a higher risk of overweight and obesity [29]. Children living with two parents may, on average, have greater access to resources and, therefore, may be at lower risk of obesity because they have consistent access to resources acquired by two adults rather than just one. Even this question is complex, however, as some research suggests that these advantages are most likely to accrue to children living with stably married, biological parents, as some stepparents decline investment in children to whom they are not biologically related or have resource obligations to biological children with whom they do not co-reside.

Exposure to family stressors, another factor that is unequally distributed across family structures, is also associated with child obesity [30]. Both adults and children in alternative family structures experience a variety of stressors that accompany family transitions and that may influence child weight, such as parental exit or entrance or residential mobility [31]. This may even be true for children whose biological parents marry after their birth, as family dynamics and space change around them. Stressors may be especially prevalent, however, for children whose biological parents split or remarry, circumstances that are often associated with family conflict [32].

Family structure selectivity presents another possible explanation for why family structure may influence child obesity. Family structure selectivity refers to how parents come to create particular family structures; for example, in the US, marriage is increasingly becoming limited to wealthier or more educated individuals [33]. These factors are also linked to child obesity [34,35]. It is possible that obesity risk is not associated with family structure, but rather that less educated parents with fewer resources, which are conditions themselves that are associated with higher childhood obesity risk, are more likely to form family structures associated with instability.

Finally, the spectrum of obesogenic factors, such as parent supervision, diet, physical activity, and screen time, may vary across family structures; for example, single parents and stepparents report less supervision of child free time than two-biological parent families [20,29,36,37]. As a result, children in these family structures may be more likely to ingest high-calorie, low-nutrition foods while unsupervised or may be less likely to receive encouragement to engage in physical activity. In fact, children in single-parent and stepparent families are also less likely to participate in formalized sports activities that require physical activity, perhaps raising their obesity risk [38].

Indeed, while some research comparing only two-parent and single-parent family structures suggests that single-parent family structures are associated with greater childhood obesity risk [23], work that examines more finely measured family structures, including cohabiting families and families in which biological parents marry after their child’s birth, finds more complex patterns. While children living with stably married biological parents had the lowest rates of obesity, this was not related to when their parents married (in other words, following the “traditional” path of the parents marrying before the child’s birth was immaterial to child obesity); in addition, children in stable cohabiting and single-parent families were no more likely to be obese, suggesting greater importance for stability than structure [14,15,16].

Understanding these competing perspectives, and the ways potential nuances across family structures and trajectories can illuminate those perspectives, is important because the potential policy interventions targeted toward families by each perspective are quite divergent. If living in nontraditional family structures is uniquely associated with childhood overweight or obesity, policy measures to fight the childhood obesity crisis might benefit from mechanisms designed to help parents marry and stay married. However, if family structure is merely a proxy for resources, stressors, selectivity, or obesogenic factors, family structures’ association with child weight could be accounted for—and perhaps even reversed—by increasing the physical, social, and emotional resources for at-risk families [39,40]. In addition, if trajectory stability is more important than family structure, interventions might be best directed toward children whose families are undergoing changes, regardless of the nature of the change.

We contribute to the current literature in three different ways. First, we look at a broader range of family structures than is typically used in such inquiries, looking at eight variations of married, cohabitating, and single-parent family trajectories that take into account both family structure and instability. Second, we add to previous examinations of the associations between family structure and overweight/obesity by testing for associations between family structure and obesogenic factors, including diet and exercise. Third, we employ a cross-national approach using data from Australia, the United Kingdom, and the United States to compare how cultural norms and social safety nets might help to explain the relationship between family structure and child weight.

## 2. Materials and Methods

### 2.1. Data

We use three datasets from Australia, the UK, and the US. Each of these datasets is nationally representative for children born in their respective countries in the initial study year. We examine children who were born near the turn of the century. This time period is two decades into the recognized period of widespread childhood obesity in western countries, when governments had already made considerable investments in considering child weight [1], as well as being three decades into demographic shifts in western countries associated with more children living outside of the traditional nuclear family [13]. Children in all three datasets were sampled on either birth (Australia and the UK) or school enrollment (the US). We excluded a very small number of children whose parents reported that their child has a serious illness or disability. This included an extremely small number of children (fewer than five per dataset) whose parents reported they had a comorbidity that might be associated with obesity, like diabetes.

The data for Australia come from Growing Up in Australia: The Longitudinal Study of Australian Children (LSAC), a dataset first collected starting in 2003 at the children’s infancy and that has followed participants every two years since (*n* = 5107) [41]. The data for the United Kingdom come from the UK Millennium Cohort Study (MCS), which follows children born between 2000 and 2002 approximately every three years (*n* = 18,818) [42]. Our United States data come from the US Early Childhood Longitudinal Study, Kindergarten Class of 1998–1999 (ECLS-K), or children born around 1994 (year varies by child and location, as state laws for age at school enrollment vary). These data track children from kindergarten through the 8th grade (*n* = 21,260) [43]. For the ECLS-K, we use retrospective data from parents about the family structure at the time of the child’s birth, allowing us to examine differences in family structure as the children age. For our outcome variable concerning weight, we use data from sweep 5 in MCS and wave 6 in LSAC and ECLS, corresponding to a child age averaging around 11 years old. We limit our samples to children with reliable measures of family structure and bodyweight categories, resulting in a total sample size of 3329 children from Australia, 11,542 children from the UK, and 8837 children from the US.

### 2.2. Measures

#### 2.2.1. Child Weight Classifications

Child bodyweight is a three-category variable that is constructed from child weight and height reports. We use these measures to create BMI values, which were then collapsed into our final weight categories as follows: not overweight, overweight, and obese (reference group = not overweight). Overweight and obese cutoffs based on BMI vary according to the age and sex of the child, so we follow traditional overweight and obesity classifications from the International Obesity Task Force [44]. For our 11-year-old boys, the overweight category includes children whose BMI scores are between 20.55 and 25.09; the obese category includes children whose BMI scores are 25.1 or greater. For our 11-year-old girls, the overweight category includes children whose BMI scores are between 20.74 and 25.41; the obese category includes children whose BMI scores are 25.42 or greater. Data for some children were gathered after they had turned 11; for children who were closer to 11.5 years old, for boys, the overweight category includes children whose BMI scores are between 20.89 and 25.57, and the obese category includes children whose BMI scores are 25.58 or greater. For girls who were close to 11.5 years old, the overweight category includes children whose BMI scores are between 21.20 and 26.04; the obese category includes children whose BMI scores are 26.05 or greater. A small number of children in each sample had not yet reached their eleventh birthdays; we assigned these children to categories based on the International Obesity Task Force’s categorization for 10.5-year-olds. For these boys, the overweight category includes children whose BMI scores are between 20.2 and 24.56; the obese category includes children whose BMI scores are 24.57 or greater. For girls in this age group, the overweight category includes children whose BMI scores are between 20.29 and 24.76; the obese category includes children whose BMI scores are 24.77 or greater. There were too few underweight children in each dataset to derive reliable estimates for underweight children, so we include them in the not overweight category. Each child, then, is assigned to a weight category (not overweight, overweight, or obese) based on how their BMI scores falls into these International Obesity Task Force categories [44]. The majority of children in each country fall into the not overweight category (see Table 1 for descriptives of all measures included in the models).

#### 2.2.2. Family Structure and Disruptions

We construct family structures and trajectories using household rosters and parent responses. We use the main parent’s relationship to the child, the main parent’s marital status, and relationship of their main parent’s partner to the child. Baseline family structure categories share some approaches to those in previous work (cf. [14,15,16]), although we add new categories to capture more diversity in biological relationships and legal recognition of couple relationships. Three of these family structure categories capture stability over the life course of the children as follows:

*Biological Married Stable*: the child lives with both biological parents, who married before the child was born.

*Biological Cohabiting Stable*: the child lives with both biological parents, who began cohabiting before the child was born.

*Biological Single Stable*: the child lives with one biological parent, who has been single since the child’s birth.

The other family structure categories indicate change or instability as follows:

*Post-Birth Biological Married Family*: the child lives with both biological parents, who married after the child was born.

*Post-Birth Stepfamily*: the child lives with one biological and one non-biological parent, who married after the child was born.

*Post-Birth Biological Cohabiting Family*: the child lives with both biological parents, who began cohabiting after the child was born.

*Post-Birth Social Family*: the child lives with one biological and one non-biological parent, who started cohabiting after the child was born.

*Post-Birth Transition to Single*: the child lives with one biological parent, who became single after the child’s birth. This includes parents who were divorced, separated, or widowed.

In our analyses, biological married stable families are the reference group.

#### 2.2.3. Explanatory Variables

We include four theoretical blocks of explanatory variables that might explain associations between family structure and a child being obese or overweight; these include measures of resources, stress, selectivity factors, and obesogenic factors.

##### Resources

Resources are measured through family income and parental employment, as low resources and having a working mother both increase the likelihood of a child being [22,36,45,46]. Income was originally measured in each country’s currency, with the UK reporting in quintiles. To facilitate cross-country comparisons, we converted the income variables in Australia and the US from their original categories to quintiles (1 = lowest, 5 = highest; reference group = lowest). Parental employment is captured through maternal and paternal employment status (reference group = full-time).

##### Stressors

We include a measure of maternal depression, as mothers in single-parent families experience increased risk for heightened stress, and children who are exposed to greater stressors, such as maternal depression, are more likely to have weight problems [31,46,47,48]. Our maternal depression scale is made up of questions that capture depressive symptoms, such as whether the mother felt nervous, restless, or worthless. The scale in Australia and the UK comes from version six of the Kessler Psychological Distress Scale, while the US scale derives from the Center for Epidemiological Studies Depression Scale (CES-D). This depression scale ranges from 0 to 24 in Australia (α = 0.85), 0 to 24 in the UK (α = 0.90), and 0 to 42 in the US (α = 0.90). We standardized the scale in each dataset to facilitate cross-country comparisons. In addition to maternal depression, we also include variables that capture family stress. The Australian data include a stress index that measures 25 stressors a family might experience, such as a major financial crisis or chronic illness; this scale has a theoretical range from 0 to 25, although no respondent reported more than 16 stressors, so in practice the range is 0–16 (α = 0.67). In the UK and US data, there is no equivalent scale, so instead, we include the following two binary variables that measure stressful scenarios a family may be exposed to: being evicted from their home and a parent unsuccessfully looking for work (reference groups = not evicted; not looking for work).

##### Family Structure Selectivity

We also explore family structure selectivity factors that may be related to an individual’s likelihood of selecting into certain family structures, which may account for differences in child obesity across family structures and trajectories. These selectivity factors measure parent demographics, including parental immigration status, parental education, and mother’s age at birth. In addition to being related to how families form, immigrant status is associated with weight differences in children, and higher levels of education are often associated with lower levels of obesity [21,47,49]. Immigration status measures whether the parents are immigrants (0 = neither are immigrants, 1 = one is an immigrant, 2 = both are immigrants; reference group = neither are immigrants). Parental education captures the highest level of education reported by either of the parents living with the child (1 = secondary school or less, 2 = some college, 3 = first postsecondary degree, 4 = higher degree; reference group = secondary school or less). Mother’s age at birth is measured in years.

We also account for other child-level factors that are commonly associated with being overweight, such as child sex, child age, child’s birth weight, and whether the child was a preterm birth [50]. Child sex is a binary variable (reference group = female). Child age is measured in years. Child race is a categorical variable (AUS: no measures of race; UK: 1 = White, 2 = Black, 3 = Asian, 4 = other; US: 1 = White, 2 = Black, 3 = Hispanic, 4 = Asian, 5 = other; reference group = White). We note that these categories reflect racial and ethnic differences across contexts; the UK data do not include a category for Hispanic but include finer measures within other categories (for example, Afro-Caribbean or African origins among Black people or additional categories within the Asian subgroup). Supplemental analyses revealed no significant differences across the racial subgroups in the UK, so we code them here to the closest match to the US data. There are no race data available in the Australian data. Birthweight is measured in ounces, and preterm birth is a binary measure (reference group = not preterm). Number of siblings has previously been shown to be related to the likelihood of being overweight [51]; in addition, disrupted families may have fewer children, or blended families may have larger numbers of children when including step and half-siblings. We measure number of children here as a count variable.

##### Obesogenic Factors

We consider a set of obesogenic factors, including sleep, sugar intake, and exercise habits, as less sleep, less physical activity, and poorer eating behaviors are associated with increased weight in children [19,51,52]. We also speculate that parental supervision patterns may vary across family trajectory, allowing for different levels of child obesogenic activity. Sleeping behavior is measured through parent reports of whether the child has a regular bedtime (reference group = does not have a regular bedtime). Sugar intake is measured by how often the child drinks sugary drinks, such as soda or juice, in a week. There are slight variations in how this variable is measured in each country, categories ranging from 0 to 3–6 sugary drinks a week in Australia to 0 to 7 or more in the UK and the US. Physical activity is captured through a combination of how often the child exercises, watches TV, and plays video or computer games. In Australia and the UK, these measures were reported by parents, while in the US, they were reported by the child. Exercise is measured through how many days a week the child exercises or participates in some sort of physical activity, ranging from one or fewer days a week (1) to five or more days a week (5). TV watching is measured in hours per day, ranging from one hour or less (1) to five or more hours a day (4). Video games captures the hours a day the child spends playing video or computer games, ranging from less than one hour a day (1) to two or more hours per day in the UK/US and three or more hours per day in Australia (3).

### 2.3. Analytic Plan

We first report descriptive statistics for each country, including means and proportions for each variable. We then compare the proportion of children in each child weight category by family structure to determine if child weight patterns differ across family and country contexts. Finally, we run a nested multinomial logistic regression with theoretical blocks for resources, stressors, family structure selectivity factors, and child-level variables to determine any associations between family structure and child bodyweight net of controls. The results of these models are presented in relative risk ratios. There were missing data in each country; on the variable with the most missing data (which varied by country), there was as much as 17% missing in Australia, 12% in the UK, and 25% in the US. We preserve cases and account for missing data using the chained equations method in Stata 16 [53]. We created 20 imputed datasets, each separated by 10 iterations as indicated by appropriate diagnostics [54]. Post-imputation tests suggest the data on missing cases are appropriate approximations.

## 3. Results

The majority of children in each country are in the not overweight category. A higher proportion of children in Australia and the UK are in the not overweight category than in the US (73 percent of children in Australia and 70 percent in the UK, compared to 61 percent in the US). The smallest proportion of children in each country are in the obese category, with the US reporting a higher proportion of obese children than Australia or the UK (14.6 percent of children in the obese category in the US compared to 6.6 percent in Australia and 7.9 percent in the UK). Still, more than 20 percent of children in each country fall into one of the overweight categories, and more than a third in the US (26.4 percent of children in either the overweight or obese category, 30.4 percent in the UK, and 37.9 in the US), indicating substantial patterns of childhood weight issues in each country by age 11.

We find that most children reside in stably married households; interestingly, this is truer in Australia and the US than in the UK. The second most common family structure is post-birth transition to single, with the UK and the US reporting more children in this family structure than in Australia. Proportions for other trajectories were generally small, with small differences across countries. As an example, Australia has fewer children in post-birth biological married families than the UK and the US but more children in biological stable cohabiting families. Overall, most children live with two parents, although across a variety of family trajectories (89% in Australia, 77% in the UK, and 85% in the US). A smaller but still substantial proportion of children have lived in stable family trajectories since birth, regardless of the number of parents with whom they have lived (83% in Australia, 58% in the UK, and 59% in the US).

In terms of our potential explanatory variables, more mothers work full-time in the US, and a substantial proportion of fathers in the UK are not in the paid labor force. More parents in Australia and the US have exposure to higher education than in the UK, and more parents are immigrants in Australia and the US, as well. Incidents of preterm birth and low birthweight are similar across countries, as are number of siblings. Children in Australia drink sugary drinks less often, watch somewhat less TV, and play video games for a shorter amount of time than their peers in the other two countries, although children in the US report more exercise (recall, however, that this is a self-reported variable, while exercise was reported by parents in the other two countries).

The descriptive comparison of the proportion of children in each weight category by family trajectory (Table 2) demonstrates that children in the US are more likely to be overweight and obese in all family structures than children in Australia or the UK. Across all three countries, stable married families consistently have the highest proportions of children who are neither overweight nor obese. Other family structures that have high rates of children who are not overweight include stable biological cohabiters, post-birth stepfamilies, and post-birth social families. Across all three countries, stably single-parented families and post-birth biological cohabiting families have the highest rates of children who fall into the obese category. There are also some differences across countries; fewer than half of the children in post-birth biological cohabiting families in the US are not overweight, whereas this proportion is closer to 70 percent in both Australia and the UK. Overall, there is evidence for both stability perspectives and structure perspectives explaining connections between child overweight/obesity and family circumstances.

Table 3, Table 4 and Table 5 present the multinomial logistic models predicting child overweight and obesity. We find that in Australia (Table 3), there were no differences in odds of being overweight in Model 1 between stably married families and other family types, although two family structures—biological cohabiting stable and biological single stable—were significantly more likely to have children who were obese. Our subsequent regression models include our theoretical blocks, which include resources, stress, selectivity, and obesogenic factors, with the final model including all explanatory variables. Even in the presence of these controls, we find an association between family structure and child obesity in Australia for children in biological cohabiting stable families, who have a higher likelihood of being obese than children in married stable families, regardless of other explanatory variables (OR: 1.609, 95% CI: 1.00, 2.57). Income, parental education, and screen time explain the initial association we saw between biological single stable families and childhood obesity.

Initial findings in the UK are more complex than in Australia (Table 4). We find in Model 1 that children in post-birth biological married and post-birth transition to single families were more likely to fall into the overweight category in the UK. Additionally, children in the following four family structures had an increased likelihood of being obese: biological single stable, post-birth biological married, post-birth biological cohabiting, and post-birth transition to single. When adding controls for our theoretical blocks, we find that children in post-birth biological married families have an increased likelihood of being both overweight and obese, even in the presence of our control variables (overweight: OR: 1.221, 95% CI: 1.02, 1.46; obese: OR: 1.329, 95% CI: 1.02, 1.73). Additionally, children in the UK in post-birth biological cohabiting families also had an increased likelihood of falling in the obese category (OR: 1.606, 95% CI: 1.01, 2.56). A few of the family structures that were originally found to be associated with an increased likelihood of obesity or overweight in Model 1 (biological single stable and post-birth transition to single) were no longer expected to have an increased likelihood once family resources were taken into account. In the UK, then, instability seems to be a more likely explanation for any connection between family structure or trajectory and child overweight/obesity.

Family structure’s association with overweight and obesity in the US seems to operate more like it does in the UK than in Australia (Table 5). Model 1 indicates that children from biological single stable, post-birth stepfamily, post-birth biological cohabiting, and post-birth transition to single families were more likely to be overweight. Additionally, children in the following four family structures had an increased likelihood of being obese: biological single stable, post-birth biological married, post-birth biological cohabiting, and post-birth transition to single. When looking at the remaining models in the US, the results indicate that children in post-birth biological cohabiting families are more likely to be overweight and obese than children in married stable families are, even in the presence of additional explanatory variables (overweight: OR: 2.516, 95% CI: 1.56, 4.05; obese: OR: 2.912, CI: 1.80, 4.72). However, the rest of the family structures that originally appeared to be related to child weight in Model 1 are no longer significantly different from the married stable comparison group once the other variables are included. The original association appears to be explained through both resource and family structure selectivity variables. While more children, and children across a wider array of family structures, fall into the overweight and obese categories in the US, children in the US also appear to be more sensitive to the effects of resources and family structure selectivity factors than children in Australia and the UK. This is particularly interesting given that social safety nets in the UK and, especially, in Australia are more robust than those in the US. The additional sensitivity to such factors in the US may reflect their relative scarcity in that country.

Across all three countries, control variables operate as expected. For example, in each country, TV time is significantly associated with child obesity, and higher birthweight is associated with an increased likelihood of being both overweight and obese. Contrary to what we might have expected, mother’s employment does not appear to be associated with child weight, with the exception of children whose mothers work part-time in the US, who are less likely to be obese than children whose mothers work full-time. Hispanic children who reside in the US and Black children who reside in the UK are more likely to be overweight or obese compared to their white counterparts.

There are also some unique country trends; for example, education appears to matter the most in the US, where higher levels of education are associated with a decreased likelihood of being overweight or obese. This trend exists in Australia and the UK, as well, but only reduces the likelihood of being obese for children whose parents have obtained higher than a postsecondary degree. This is particularly notable given the findings above that family selectivity variables were most important for explaining patterns for children in the US. In the UK, income is the most important factor, while in the US, both income and parental education are key. In Australia, we must consider resources, family structure selectivity, and obesogenic factors (TV watching) to render initially significant associations between living in a stably single-parent family and obesity nonsignificant. Still, even when taking these factors into account, we find patterns of obesity risk associated with family structures, particularly structures with cohabiting parents and trajectories of family instability across the three country contexts.

We also investigated whether any explanatory factors among resources, stressors, selectivity, or obesogenic factors exhibited stronger effects (e.g., more pronounced slopes) for some family structures. For example, it is possible that stressors might operate more strongly in family structures characterized by instability than in nontraditional but stable family structures. To perform these tests, we ran interactions for each country exploring whether the potential effects of resources, stressors, family structure selectivity factors, or obesogenic behaviors varied by family structure. We found no significant interaction effects in any of the three countries, suggesting that the effects of explanatory variables on child overweight or obesity operate in similar fashion across different family structures and trajectories.

## 4. Discussion

We expected that family structure and family instability would play a significant role in predicting a child’s likelihood of being either overweight or obese. However, we found that most of this association can be explained by family resources and family structure selectivity factors. Despite this, there were a few family structures whose relationship with child weight persisted even in the presence of other explanatory factors. In the UK and the US, it appears that there is something important about parents who have a child before entering into a union together, whether it be cohabitation or marriage, as their children are more likely to be overweight. This is surprising considering the evidence that children who live with both biological parents are generally expected to have better outcomes, but post-birth entrances to such contexts were associated with an increased likelihood of overweight or obesity in our data see also [20]. Future research should investigate this relationship further to determine what is unique about biological parents who enter a cohabiting or marital union after the birth of their child. These patterns indicate a potential pathway for interventions that could prevent child overweight and obesity. Physicians, social workers, and similar practitioners should ask questions about adults entering or leaving households and could provide education on healthy eating and exercise behaviors to families in which birth fathers legally enter the family after their child’s birth.

By contrast, in Australia, children residing in biological cohabiting stable families are more likely to be obese than children in married stable families. Given coefficient sizes and the finding that all forms of cohabiting unions in Australia are more likely to have children that are obese, we suspect that the non-significance of the other cohabiting family structures may be due to the relatively small number of children in these family structures. Biological cohabiting stable families are relatively common in Australia compared to the other two countries we examine here, so understanding and tailoring interventions for these families could be an important way to decrease child obesity in Australia. We speculate that normalizing cohabitation in Australia could decrease the likelihood of children in these family structures being overweight or obese.

Additionally, although marriage incentives may seem like promising policy interventions, our results provide evidence that simply being married does not substantially decrease the child’s likelihood of being overweight or obese. If this is a case of selectivity, we would suggest that policymakers focus less on the marital status of parents and more on their educational status. Helping parents in these family structures obtain further education and greater access to resources would likely increase the overall stability in their homes, in turn further mitigating any association between family structure and their child’s likelihood of being overweight or obese.

### Limitations

Although we measure a larger number of family structures and trajectories to attempt to account for greater variation in children’s experiences than is found in previous research, this study has a number of limitations to consider. One difficulty in using otherwise desirable large, nationally representative datasets, as we have here, is that data from the different countries were not gathered by the same body or using all the same instruments. We made it a priority to harmonize variables and concepts across the three countries to provide better comparisons, but optimal harmonization was not always feasible. For example, the stress measure for Australia is a multifactor scale, while stress could only be measured with two similar items in the UK and US data, making direct comparisons difficult. In addition, there are no measures for race or ethnicity available in the Australian data, despite that country’s relatively high in-migration rate, especially from Asian countries. It would have been desirable to conduct multilevel models as a direct test of whether slopes on key variables differ across countries, but this was not possible because each dataset was independent of the others. It is also true that there were relatively small proportions of some of the nontraditional family structures, especially in the smaller Australian sample. We ran sensitivity tests and power analyses that increased our confidence that the number of cases in those smaller categories was sufficient for the statistical models we employ here, but we note data with larger samples of those less common family structures might produce different patterns of findings.

We also note that there were some potential explanatory or confounding factors that we could not explore here due to data limitations. For example, none of the datasets include information on parents’ weight or BMI. While we argue that robust social safety nets in the UK and, especially, Australia may provide protection for at-risk children that children in the US do not enjoy, we are not able to measure the specific use of most public programs. For example, there was no variance in the uptake of financial payments from the government to parents in the UK data. The UK data provided some variables on other government programs; additional tests using such variables revealed no substantial differences. However, we were not able to test these ideas in the Australian or US data, and it is possible that more detailed social support variables might be associated with less risk of child overweight/obesity across different family structures. We also lack data on either financial or social support from non-residential parents. We are also unable to establish relationships with parental gender. Most respondent parents across all three countries are mothers, and there are insufficient numbers of, for example, single-father families to draw any conclusions about children living only with fathers. Similarly, there are too few same-sex couples among the data to be able to draw any conclusions about how those family structures might be associated with child overweight/obesity. We would have preferred to measure physical activity in hours per week to make more granular conclusions about activity, but the datasets we use here only provide information for physical activities in days per week. Finally, we acknowledge that some scholars argue that BMI is a poor measure of obesity and a poor proxy of actual health conditions [55]. While we follow the general literature on child weight by using BMI and BMI cutoffs here, we hope that future research will examine a wider range of measures of adiposity, body size and shape, and socially constructed definitions of fatness and obesity among children. Although we encourage readers to take these potential limitations into account while considering the findings we present here, we believe that the robust models we test, the multiple theories we examine, the large datasets, and the consistency of explanatory effects across countries increase confidence in the findings we report here. Finally, we note that the children we studied here were age 11 between the years of 2006 and 2011; it is possible that the obesity crisis has increased since that time. On the other hand, it is possible that successful intervention policies have been introduced since that time or that the cultures in these countries have become more accepting of alternative family structures since this time, potentially leading to less childhood overweight and obesity. Reports on 2023 data suggest slightly higher rates of childhood obesity in these countries than in 2011 [56], however, so we suspect that the findings we report here continue to reflect contemporary experiences of family trajectories and childhood overweight and obesity.

## 5. Conclusions

Much of the literature surrounding child obesity and family structure suggests that obesogenic factors, such as access to healthy meals, limited sugar intake, encouragement to exercise, and parent supervision can be used to explain the prevalence of child obesity among different family structures [29,36]. However, we find stronger ties between family structure and resources or family structure selectivity factors than to obesogenic behaviors. Blaming, for example, single parents for being less available to monitor screen time or snacks may not be an effective way to address the childhood obesity crisis. Rather, across all three countries we study here, giving additional resources directly to families in the form of money and education seems more salient. The importance of race in the UK and US provides additional evidence that addressing systemic inequality may contribute more to stemming the childhood obesity crisis than pointing to individual families’ choices. Overall, we suggest that future research explores the association with resources and selectivity factors that can have on addressing the issue of childhood obesity.

## Figures and Tables

**Table 1:** Descriptives table.

Variable	Mean/Proportion			Standard Deviation			Range
	Australia	UK	US	Australia	UK	US	Australia, UK, US
Child Weight (proportion of sample)							1–3
Not Overweight	0.730	0.696	0.621				
Overweight	0.204	0.225	0.233				
Obese	0.066	0.079	0.146				
Family Structure (proportion of sample)							1–8
Biological Married Stable	0.716	0.481	0.539				
Biological Cohabiting Stable	0.082	0.040	0.004				
Biological Single Stable	0.028	0.054	0.042				
Post-Birth Biological Married	0.047	0.112	0.133				
Post-Birth Stepfamily	0.011	0.055	0.070				
Post-Birth Biological Cohabiting	0.008	0.021	0.021				
Post-Birth Social Family	0.024	0.059	0.024				
Post-Birth Transition to Single	0.083	0.178	0.166				
Income (reported in quintiles)							1–5
Bottom	0.188	0.192	0.249				
Second	0.200	0.201	0.214				
Third	0.208	0.211	0.202				
Fourth	0.200	0.206	0.151				
Top	0.203	0.190	0.185				
Mother’s Employment (proportion of sample)							1–3
Full-time	0.397	0.211	0.504				
Part-time	0.397	0.465	0.242				
Not in paid labor force	0.206	0.324	0.255				
Father’s Employment (proportion of sample)							1–3
Full-time	0.872	0.599	0.738				
Part-time	0.053	0.091	0.033				
Not in paid labor force	0.075	0.310	0.229				
Maternal Depression Scale (standardized)	0.002	0.001	0.002	0.000	0	0.000	
Stress Scale (standardized)	2.421	-	-	2.281	-		0–16
Evicted (1 = yes)	-	0.005	0.001				0–1
Looking for work (1 = yes)	-	0.038	0.031				0–1
Highest Parent Education (proportion of sample)							1–4
Secondary school or less	0.302	0.544	0.250				
Some college	0.134	0.155	0.343				
First postsecondary degree	0.252	0.194	0.210				
Higher degree	0.312	0.106	0.198				
Parent’s Immigration Status (proportion of sample)							0–2
Neither parent is an immigrant	0.658	0.797	0.614				
One parent is an immigrant	0.238	0.054	0.092				
Both parents are immigrants	0.104	0.149	0.294				
Child Sex (1 = male)	0.511	0.505	0.498				0–1
Child Age (years)	10.956	11.164	11.226	0.063	0.003	0.005	10–13
Child Race (proportion of sample)							N/A, 1–4, 1–5
White	-	0.851	0.613				
Black	-	0.028	0.096				
Hispanic	-	-	0.181				
Asian	-	0.090	0.057				
Other	-	0.031	0.053				
Preterm Birth (1 = yes)	0.075	0.075	0.064				0–1
Birth Weight (ounces)	118.878	118.873	119.121	0.345	0.206	0.220	16–255
Number of Siblings	1.612	1.569	1.56	0.018	0.010	0.012	0–10, 0–10, 0–12
Child has regular bedtime (1 = yes)	0.926	0.898	0.927				0–1
Juice/soda consumption (proportion of sample)							1–3, 1–4, 1–4
0 times a week	0.332	0.400	0.152				
1–2 times a week (1–3 in US)	0.515	0.210	0.382				
3–6 times a week (4–6 in US)	0.153	0.085	0.172				
7+ days a week	-	0.305	0.294				
Physical Activity (proportion of sample)							1–5
0–1 days a week	0.226	0.190	0.035				
2 days a week	0.183	0.130	0.044				
3 days a week	0.203	0.116	0.08				
4 days a week	0.142	0.079	0.136				
5+ days a week	0.247	0.484	0.706				
TV Watching (proportion of sample)							1–4
0–1 h a day	0.176	0.167	0.282				
1–3 h a day	0.663	0.684	0.300				
3–5 h a day	0.121	0.106	0.245				
5+ h a day	0.041	0.043	0.173				
Video Games (proportion of sample)							1–3
Less than 1 h a day	0.797	0.541	0.423				
1–2 h a day (1–3 in AUS)	0.185	0.306	0.274				
More than 2 h a day (3+ in AUS)	0.019	0.154	0.302				

Note: Australia *n* = 3329; United Kingdom *n* = 11,542; United States *n* = 8837.

**Table 2:** Proportion of children in body weight category by family structure in Australia, the UK, and the US.

	Biological Married Stable	Biological Cohabiting Stable	Biological Single Stable	Post-Birth Biological Married	Post-Birth Stepfamily	Post-Birth Biological Cohabiting	Post-Birth Social Family	Post-Birth Transition to Single
Australia								
Not Overweight	0.75	0.73	0.62	0.74	0.69	0.69	0.72	0.64
Overweight	0.19	0.17	0.24	0.20	0.23	0.21	0.22	0.27
Obese	0.06	0.10	0.14	0.06	0.08	0.10	0.06	0.09
UK								
Not Overweight	0.72	0.70	0.62	0.67	0.73	0.66	0.70	0.65
Overweight	0.21	0.23	0.25	0.24	0.21	0.22	0.23	0.24
Obese	0.07	0.07	0.13	0.09	0.06	0.12	0.07	0.11
US								
Not Overweight	0.66	0.58	0.53	0.57	0.61	0.45	0.61	0.57
Overweight	0.22	0.29	0.24	0.25	0.25	0.29	0.22	0.25
Obese	0.12	0.13	0.23	0.18	0.14	0.26	0.17	0.18

Note: Australia *n* = 3329; United Kingdom *n* = 11,542; United States *n* = 8837.

**Table 3:** Multinomial logistic regression of child weight on family structure, stress, selectivity, obesogenic factors, and controls in Australia (*n* = 3329).

Variable	Overweight						Obese					
	Model 1	Model 2	Model 3	Model 4	Model 5	Model 6	Model 1	Model 2	Model 3	Model 4	Model 5	Model 6
Family Structure												
Biological Cohabiting Stable	0.951	0.902	0.950	0.894	0.901	0.839	1.844 *	1.672 *	1.859 **	1.626 *	1.786 *	1.609 *
Biological Single Stable	1.234	1.018	1.216	1.086	1.149	0.935	2.188 *	1.473	2.116 *	1.896	1.955	1.417
Post-Birth Biological Married	0.999	0.949	1.001	0.944	0.951	0.902	1.065	0.969	1.072	0.934	0.924	0.844
Post-Birth Stepfamily	1.393	1.268	1.341	1.404	1.345	1.279	0.886	0.706	0.817	0.882	0.896	0.847
Post-Birth Biological Cohabiting	2.312	2.182	2.269	2.434	2.241	2.388	2.494	2.030	2.42	2.247	2.358	2.108
Post-Birth Social Family	1.211	1.071	1.208	1.191	1.167	1.096	1.474	1.198	1.493	1.353	1.481	1.350
Post-Birth Transition to Single	1.175	0.969	1.166	1.067	1.152	0.936	1.316	0.940	1.308	1.115	1.324	0.967
Resources												
Income												
Second		0.908				0.949		0.740				0.795
Third		0.699				0.732		0.481 **				0.562 *
Fourth		0.709				0.786		0.397 **				0.501 *
Top		0.513 **				0.588 *		0.441 **				0.658
Mother’s Employment												
Part-time		0.813				0.857		0.756				0.837
Not in paid labor force		0.766				0.845		0.889				0.943
Father’s Employment												
Part-time		1.13				1.158		0.924				0.947
Not in paid labor force		0.924				0.966		0.999				1.051
Stressors												
Maternal Depression			0.945			0.963			0.861			0.921
Family Stressor Scale			0.991			0.988			0.96			0.949
Selectivity												
Highest Parent Education												
Some college				0.899		0.947				0.682		0.734
First postsecondary degree				0.694		0.793				0.497 **		0.646
Higher degree				0.677		0.812				0.389 ***		0.518 **
Parent’s Immigration Status												
One parent is an immigrant				0.969		0.957				0.852		0.826
Both parents are immigrants				1.185		1.134				1.369		1.185
Child Sex				0.831		0.82				1.23		1.263
Child Age				0.912		0.922				1.023		0.997
Mother’s Age at Birth				0.999		1.002				0.998		0.999
Preterm				1.321		1.289				0.988		0.96
Birth Weight				1.012 ***		1.012 ***				1.007		1.007
Number of Siblings				0.865 *		0.864 *				0.946		0.929
Obesogenic Factors												
Child has regular bedtime					0.823						0.624	0.643
Juice/soda consumption												
1–2 times a week					1.037	1.003					1.161	1.108
3–6 times a week					1.374 *	1.334					1.436	1.267
7+ days a week					-	-					-	-
Physical Activity												
2 days a week					1.058	1.121					0.920	0.982
3 days a week					0.961	0.963					1.037	1.083
4 days a week					1.160	1.183					1.102	1.202
5+ days a week					0.974	1.029					0.597 *	0.635
TV Watching												
1–3 h a day					1.414 *	1.327 *					3.225 ***	2.907 **
3–5 h a day					1.459 *	1.289					3.559 ***	2.938 **
5+ h a day					1.298	1.228					3.835 **	3.448 **
Video Games												
1–3 h a day					1.077	1.059					1.504 *	1.512 *
More than 3 h a day					0.869	0.89					0.776	0.796

Note: Model 1 includes family structure (FS), Model 2 includes FS and resources, Model 3 includes FS and stress, Model 4 includes FS and selectivity, Model 5 includes obesogenic factors, and Model 6 includes all variables. Child race was not measured in Australian data. * *p* < 0.05. ** *p* < 0.01. *** *p* < 0.001.

**Table 4:** Multinomial logistic regression of child weight on family structure, stress, selectivity, obesogenic factors, and controls in the United Kingdom (*n* = 11,542).

Variable	Overweight						Obese					
	Model 1	Model 2	Model 3	Model 4	Model 5	Model 6	Model 1	Model 2	Model 3	Model 4	Model 5	Model 6
Family Structure												
Biological Cohabiting Stable	1.178	1.119	1.161	1.166	1.157	1.102	1.296	1.064	1.245	1.256	1.272	1.027
Biological Single Stable	1.067	0.962	1.017	1.018	1.033	0.922	2.023 ***	1.065	1.775 **	1.651 **	1.854 ***	0.876
Post-Birth Biological Married	1.261 **	1.193 *	1.239 *	1.267 **	1.270 **	1.221 *	1.524 **	1.275	1.455 **	1.502 **	1.559 **	1.329 *
Post-Birth Stepfamily	0.963	0.883	0.94	1.005	0.972	0.943	0.905	0.665	0.853	0.946	0.917	0.731
Post-Birth Biological Cohabiting	1.133	1.060	1.106	1.200	1.122	1.121	2.158 **	1.535	2.013 **	2.301 **	2.067 **	1.606 *
Post-Birth Social Family	1.076	0.986	1.045	1.122	1.079	1.042	1.193	0.852	1.103	1.245	1.215	0.916
Post-Birth Transition to Single	1.322 **	1.207	1.266	1.283 **	1.286 **	1.185	1.744 ***	1.018	1.566 ***	1.579 ***	1.624 ***	0.945
Resources												
Income												
Second		1.180				1.103		1.294				1.001
Third		1.134				1.015		1.112				0.748
Fourth		0.972				0.880		0.694 *				0.458 ***
Top		0.800				0.721 *		0.480 ***				0.305 ***
Mother’s Employment												
Part-time		0.943				0.950		0.799				0.803
Not in paid labor force		0.922				0.939		0.961				1.007
Father’s Employment												
Part-time		0.985				0.956		1.110				1.045
Not in paid labor force		1.014				0.961		1.387 *				1.19
Stressors												
Maternal Depression			1.085 **			1.065 *			1.225 ***			1.127 *
Evicted			1.497			1.405			1.197			0.922
Looking for work			0.966			0.979			0.922			0.802
Selectivity												
Highest Parent Education												
Some college				0.839		0.922				0.756		1.009
First postsecondary degree				0.763 **		0.882				0.510 ***		0.748
Higher degree				0.681 **		0.818				0.375 ***		0.591 *
Parent’s Immigration Status												
One parent is an immigrant				0.838		0.837				0.906		0.885
Both parents are immigrants				1.007		0.997				1.021		0.998
Child Sex				0.735 ***		0.761 ***				0.775 **		0.802 *
Child Age				0.878		0.88				0.734 *		0.738 *
Child Race												
Black				1.691 **		1.508 *				2.714 ***		1.990 **
Asian				1.428 *		1.267				1.896 **		1.181
Other				1.172		1.108				1.764 *		1.524
Mother’s Age at Birth				1.008		1.014 *				1.016		1.035 ***
Preterm				1.308 *		1.301				1.270		1.200
Birth Weight				1.008 ***		1.008 ***				1.009 ***		1.010 ***
Number of Siblings				0.911 **		0.894				0.866 **		0.771 ***
Obesogenic Factors												
Child has regular bedtime					0.872	0.904					0.612 ***	0.697 **
Juice/soda consumption												
1–2 times a week					0.987	0.981					1.009	0.994
3–6 times a week					1.059	1.047					1.002	0.927
7+ days a week					1.096	1.080					1.136	1.037
Physical Activity												
2 days a week					0.830	0.858					0.803	0.863
3 days a week					0.862	0.891					0.685 *	0.737
4 days a week					0.887	0.931					0.833	0.92
5+ days a week					0.718 ***	0.769 **					0.478 ***	0.522 ***
TV Watching												
1–3 h a day					1.342 ***	1.281 **					1.693 ***	1.553 **
3–5 h a day					1.784 ***	1.641 ***					2.228 ***	1.904 ***
5+ h a day					1.889 ***	1.748 ***					2.004 **	1.734 *
Video Games												
1–2 h a day					0.968	1.013					1.022	1.019
More than 2 h a day					0.820 *	0.887					1.023	1.033

Note: Model 1 includes family structure (FS), Model 2 includes FS and resources, Model 3 includes FS and stress, Model 4 includes FS and selectivity, Model 5 includes obesogenic factors, and Model 6 includes all variables. Hispanics were not measured in UK data. * *p* < 0.05. ** *p* < 0.01. *** *p* < 0.001.

**Table 5:** Multinomial logistic regression of child weight on family structure, stress, selectivity, obesogenic factors, and controls in the United States (*n* = 8837).

Variable	Overweight						Obese					
	Model 1	Model 2	Model 3	Model 4	Model 5	Model 6	Model 1	Model 2	Model 3	Model 4	Model 5	Model 6
Family Structure												
Biological Cohabiting Stable	1.271	1.055	1.243	1.084	1.121	0.980	1.372	1.038	1.358	1.100	1.238	0.979
Biological Single Stable	1.731 ***	1.407 *	1.722 ***	1.262	1.588 **	1.169	1.829 ***	1.391	1.823 ***	1.382	1.598 **	1.337
Post-Birth Biological Married	1.042	0.921	1.032	0.826	1.01	0.810	1.755 **	1.452 *	1.748 **	1.356	1.656 **	1.306
Post-Birth Stepfamily	1.307 *	1.188	1.291 *	1.203	1.239	1.152	1.048	0.895	1.047	1.061	0.988	1.006
Post-Birth Biological Cohabiting	2.925 ***	2.517 ***	2.902 ***	2.588 ***	2.838 ***	2.516 ***	3.620 ***	2.876 ***	3.613 ***	3.100 ***	3.390 ***	2.912 ***
Post-Birth Social Family	0.885	0.759	0.879	0.658	0.861	0.635	1.184	0.899	1.178	0.933	1.177	0.881
Post-Birth Transition to Single	1.329 **	1.070	1.307 **	0.957	1.249 *	0.860	1.558 ***	1.163	1.552 ***	1.160	1.395 **	1.072
Resources												
Income												
Second		0.834				0.906		0.977				1.055
Third		0.877				1.045		0.710				0.837
Fourth		0.552 **				0.688 *		0.604				0.773
Top		0.607 **				0.814		0.473 ***				0.628 *
Mother’s Employment												
Part-time		0.852				0.868		0.652 **				0.685 **
Not in paid labor force		0.834				0.862		0.781				0.823
Father’s Employment												
Part-time		0.964				0.946		0.979				0.944
Not in paid labor force		0.998				1.019		0.958				0.905
Stressors												
Maternal Depression			1.105			1.079			1.038			1.007
Evicted			1.341			1.294			0.665			0.640
Looking for work			0.794			0.810			0.944			0.892
Selectivity												
Highest Parent Education												
Some college				0.731 *		0.740 *				0.831 *		0.839
First postsecondary degree				0.486 ***		0.533 ***				0.437 ***		0.503 **
Higher degree				0.611 **		0.691				0.537 ***		0.661 *
Parent’s Immigration Status												
One parent is an immigrant				0.914		0.898				1.02		1.017
Both parents are immigrants				1.169		1.134				1.225		1.180
Child Sex				0.866		0.874				1.055		1.017
Child Age				1.159 *		1.155 *				0.770 *		0.774 *
Child Race												
Black				1.172		1.075				1.531 **		1.322 *
Hispanic				1.430 **		1.354 **				1.743 ***		1.559 ***
Asian				0.842		0.800				1.319		1.171
Other				1.185		1.103				1.730 **		1.542 *
Mother’s Age at Birth				0.996		0.997				1.017		1.020 *
Preterm				2.047 ***		2.026 ***				2.008 ***		1.970 ***
Birth Weight				1.014 ***		1.014 ***				1.018 ***		1.018 ***
Number of Siblings				0.866 ***		0.866 ***				0.912 *		0.914 *
Obesogenic Factors												
Child has regular bedtime					0.787	0.793					0.734	0.755
Juice/soda consumption												
1–3 times a week					0.978	0.991					0.893	0.927
4–6 times a week					0.966	0.946					0.771	0.792
7+ days a week					1.092	1.042					0.666 *	0.662 *
Physical Activity												
2 days a week					1.208	1.211					1.042	1.121
3 days a week					1.241	1.243					1.853	1.976
4 days a week					1.023	1.012					1.692	1.793
5+ days a week					1.025	1.057					1.430	1.537
TV Watching												
1–3 h a day					1.257	1.197					1.607 **	1.460 *
3–5 h a day					1.427 *	1.272					2.321 ***	1.985 ***
5+ h a day					1.488 *	1.342					2.172 ***	1.878 ***
Video Games												
1–2 h a day					1.141	1.162					1.092	1.075
More than 2 h a day					0.899	0.918					1.087	1.064

Note: Model 1 includes family structure (FS), Model 2 includes FS and resources, Model 3 includes FS and stress, Model 4 includes FS and selectivity, Model 5 includes obesogenic factors, and Model 6 includes all variables. * *p* < 0.05. ** *p* < 0.01. *** *p* < 0.001.

## Data Availability

Restrictions apply to the availability of these data. Australian data were obtained from the Australian Bureau of Statistics and are available from https://growingupinaustralia.gov.au/data-and-documentation/accessing-lsac-data, accessed on 15 April 2024. UK data were obtained from the Centre for Longitudinal Studies and are available from https://beta.ukdataservice.ac.uk/datacatalogue/series/series?id=2000031, accessed on 15 April 2024. US data were obtained from the National Center for Education Statistics and are available from https://nces.ed.gov/ecls/kinderdatainformation.asp, accessed on 15 April 2024.
